# Coupling of the Functional Stability of Rat Myocardium and Activity of Lipid Peroxidation in Combined Development of Postinfarction Remodeling and Diabetes Mellitus

**DOI:** 10.1155/2016/2548689

**Published:** 2015-12-30

**Authors:** S. A. Afanasiev, D. S. Kondratieva, T. Yu. Rebrova, R. E. Batalov, S. V. Popov

**Affiliations:** Federal State Budgetary Scientific Institution “Research Institute for Cardiology”, 111a Kievskaya Street, Tomsk 634012, Russia

## Abstract

Coupling of the functional stability of rat myocardium and activity of lipid peroxidation processes in combined development of postinfarction remodeling and diabetes mellitus has been studied. The functional stability of myocardium was studied by means of the analysis of inotropic reaction on extrasystolic stimulus, the degree of left ventricular hypertrophy, and the size of scar zone. It was shown that in combined development of postinfarction cardiac remodeling of heart (PICR) with diabetes mellitus (DM) animal body weight decreased in less degree than in diabetic rats. Animals with combined pathology had no heart hypertrophy. The amplitude of extrasystolic contractions in rats with PICR combined with DM had no differences compared to the control group. In myocardium of rats with PICR combined with DM postextrasystolic potentiation was observed in contrast with the rats with PICR alone. The rats with combined pathology had the decreased value of TBA-active products. Thus, the results of study showed that induction of DM on the stage of the development of postinfarction remodeling increases adaptive ability of myocardium. It is manifested in inhibition of increase of LPO processes activity and maintaining of force-interval reactions of myocardium connected with calcium transport systems of sarcoplasmic reticulum of cardiomyocytes.

## 1. Introduction

Diabetes mellitus (DM) is one of the threatening factors which increases the risk of cardiovascular accidents during cardiovascular diseases [1]. Metabolic changes developing during diabetes mellitus aggravate disorders of functional state of cardiomyocytes in heart failure (HF) [2–4]. It is caused to a great extent by change of energy metabolism, which is an additional trigger of functional and structural disorders of heart muscle. In turn, remodeling of the cardiomyocyte membranes with advanced glycation end products and free radical oxidation is essential factor in development of diabetes mellitus [5, 6]. All these factors contribute to the disorder of electrical stability of membranes and the ionic balance of heart cells. These changes may define mainly cardiomyocyte contractility. The key structure, responding to intracellular transport of Са^2+^ and, accordingly, to inotropic response of cardiomyocytes, is sarcoplasmic reticulum (SR) [7]. It has been shown that disorder of SR functions is accompanied by the inversion of force-frequency and force-interval dependences of myocardium [8, 9]. The interrelation between change of Са^2+^ homeostasis in cardiomyocytes and progression of HF is revealed: disorder of intracellular Са^2+^ transport precedes the depression of mechanical performance of heart [10–12].

An important role in disorder of ion transport systems of cardiomyocytes is played by lipid peroxidation (LPO) processes [13]. Intensification of LPO is nonspecific cell reaction to pathological actions. Development of HF and DM is accompanied with considerable increase of LPO activity [14, 15]. So, it is shown that LPO products act on lipid phase of membranes making it penetrable for hydrogen and calcium ions. It results in uncoupling of oxidative phosphorylation in mitochondria which leaves cell in the state of energy deficiency. At that state the excess amount of Са^2+^ entering the cytoplasm is not able to be withdrawn from myoplasm and, subsequently, damages cellular structures.

In contrast to clinical data, which unambiguously points to the decrease of stability of diabetic heart to ischemia, results of experimental studies are sufficiently contradictory. So, in number of researches one notes paradoxically high myocardial resistance to ischemia (in vivo and in vitro) in adult animals with short-term streptozotocin-induced diabetes [16–18]. Our preliminary study also revealed facts of the maintenance of the myocardial contractility in combined development of HF and DM. Mechanisms of this phenomenon remain subject for scientific research. States of Са^2+^ transport systems of cardiomyocyte SR and activity of LPO processes in combined development of HF and DM have been studied insufficiently.

## 2. Materials and Methods

The study was performed on adult male Wistar rats 200–220 g. Four groups of animals were formed: first group consisted of intact rats (*n* = 12), second group of the rats with postinfarction cardiac remodeling (PICR) (*n* = 11), third group of the rats with induced DM (*n* = 8), and the IV group of the rats with DM induced 2 weeks after coronary occlusion (*n* = 8). By the time of the experiment all animals were of the same age. Myocardial infarction was induced by means of occlusion of the left anterior descending artery [19]; then the animals were housed under standard vivarium conditions. Diabetes mellitus was induced by single injection of 60 mg/kg dose of streptozotocin (“Sigma,” USA) abdominally, diluted ex tempore with 0.01 M/L citrate buffer (pН 4.5). Rats of the IV group were taken in the experiment 6 weeks after induction of diabetes. Concentration of glucose in blood serum was defined by enzymatic-colorimetric test (“Biocon Diagnostic,” Germany).

The development of heart and left ventricle hypertrophy was estimated by corresponding mass ratio [20]. For that reason the ratios of heart mass to animal body mass and left ventricle mass to heart mass were defined. Size of postinfarction scars of animal heart was estimated by the method of planimetry and calculated in percentages from area of free wall of left ventricle [21].

In the day of experiment animal blood has been sampled in a tube with heparin (10 : 1). Blood samples were centrifuged at 3000 rpm for 10 min. Obtained serum was dispensed for aliquots and stored in liquid nitrogen until the investigation moment.

Contractile activity was studied on papillary muscles. For that animals under Rausch-narcosis were immobilized with displacement of cervical region of the vertebral column and then their chests were opened. Isolated heart was washed in the specialized flow chamber through aorta with Krebs-Henseleit solution of the following composition (in mM): NaCl: 120; KCl: 4.8; CaCl_2_: 2.0; MgSO_4_: 1.2; KH_2_PO_4_: 1.2; NaHCO_3_: 20.0; glucose: 10.0 (“Sigma,” USA). Then, papillary muscles were isolated and placed in the temperature-stabilized (36°С) flow chamber. Perfusion of muscles has been performed with Krebs-Henseleit solution. Oxygenation of solution has been performed with carbogen (О_2_: 95%, СО_2_: 5%). Contractile activity of muscles was estimated in isometric mode, using “Force transducer KG-Series” transducer (Scientific Instruments GmbH, Germany). Tension developed by muscle calculated on diameter of isolated muscle (mN/mm^2^) was estimated. Stimulation of muscles was performed with rectangular electrical pulses with duration of 5 ms and frequency of 0.5 Hz. Before the beginning of the research muscles had been adapted to the perfusion conditions and isometric mode in 60 minutes.

It is known that functional state of isolated myocardial strips can be estimated by changing the mode of their electrical stimulation. At extrasystolic impact, we registered extrasystolic contraction which characterizes excitability of sarcolemma [22] and postextrasystolic contraction which reflects the ability of cardiomyocyte sarcoplasmic reticulum (SR) to accumulate Са^2+^ ions which additionally enter the myoplasm at extraordinary excitation and define amplitude of postextrasystolic contractions [22]. In our work, extrasystolic impact was made by additional single electrical pulse on 0.2, 0.225, 0.25, 0.5, 0.75, 1.0, and 1.5 s (extrasystolic interval) from the beginning of the regular cycle. Amplitudes of extrasystolic (ES) and postextrasystolic (PES) contraction were expressed as percentages of the amplitude of regular (basic) cycle. We analyzed the dependence of changes of ES and PES contraction amplitude on the duration of extrasystolic interval.

LPO activity in blood serum was estimated by measuring the concentration of TBA-active products (TBAAP) acquired in reaction with 2-thiobarbituric acid (TBA) [23]. The concentration of primary products of LPO-dien conjugates (DC) was measured in the hexane extracts of serum samples with spectrophotometer at 232 nm [24].

Data is presented in the form of median and interquartile range (Мe (Q1; Q3)). Student's criterion has been used for normal distribution of values. Study data is presented as M ± SD, where M is mean value and SD is standard deviation. Reliability of differences of obtained data was estimated using Mann-Whitney *U* test for independent samples in the case of distribution which differs from normal one. Differences at value *p* < 0.05 have been considered statistically significant.

## 3. Results and Discussion

Results reflecting values of mass indices obtained in considered groups are presented in Table 1. It can be seen that the animals with PICR (the II group) had decreased (on 18.8%) body weight and hypertrophied (on 90%) heart compared to those of intact animals. Induction of diabetes (the III group) led to decreased animal body weight by 56%, *p* < 0.05, but in this case without heart hypertrophy. In combined development of PICR with DM (the IV group), animal body weight was 26% less than the one of the animals from the I group. These animals as well as the animals in the III groups did not have heart hypertrophy. It appeared that size of scar zone in II and IV groups did not differ. Blood glucose level of the animals of III and IV groups exceeded that of intact rats by 4.5 and 3 times, accordingly.

**Figure 1:**
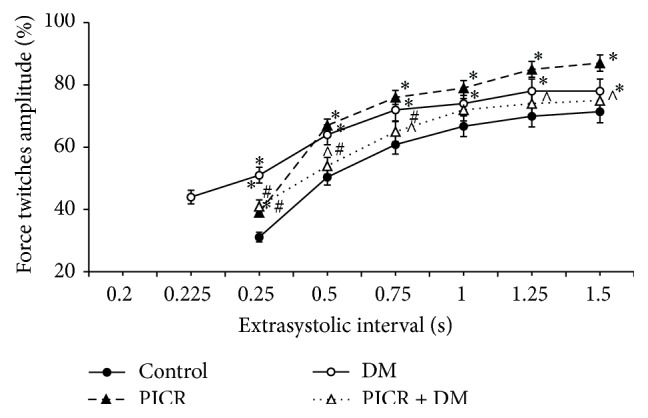
Extrasystolic contractions of papillary muscles of rats with postinfarction heart failure and diabetes mellitus. Note: the force twitches amplitude expressed in percentage of base contraction. ^*∗*^ *p* < 0.001 compared with control, ^#^ *p* < 0.05 compared with DM, and ^∧^ *p* < 0.05 compared with PICR.

In our study, the remodeling of myocardium both after coronary artery occlusion (the II group) and after development of hyperglycemia (the III group) led to a change in inotropic reaction of papillary muscles on extrasystolic actions compared to the control group (Figure 1). So, amplitude of ES contractions of papillary muscles of the PICR rats (the II group) on short extrasystolic intervals was 8% higher than that of intact animals (*p* < 0.05). After the longest ES interval, this difference increased and reached 16% (*p* < 0.05). Amplitude increase of ES contractions of papillary muscles of PICR testifies the increased intracellular amount of Са^2+^ taking part in ES contraction. It is known that ischemic damage of heart is characterized by the suppression of ATP-sensitive processes including the work of intracellular ion transport systems. It leads to the increase in intracellular concentrations of Na^+^ and Са^2+^ [25–27]. ES contractions of papillary muscles of the rats from the III group have their own peculiarities. So, independent ES contraction appeared already at ES interval of 0.225 s. In the rest of the groups ES contraction appeared only at ES interval of 0.25 s. It is known that ES action causes inotropic response only if it happens in the phase of relative refractivity [22]. From these positions result obtained in the III group shows that development of diabetes leads to a shortened phase of absolute refractivity and hence to an increased excitability of cardiomyocytes. The fact that the amplitude of ES contractions in the III group on short extrasystolic intervals was 20% higher than that in the I group (intact animals) testifies in favor of that. At long intervals these differences decreased to 7% (Figure 1).

Result obtained by studying the IV group differs from the case of II or III group. In case of combined development of ischemic and diabetic damage of myocardium we obtained essentially less manifested change of ES contraction dynamics.

**Figure 2:**
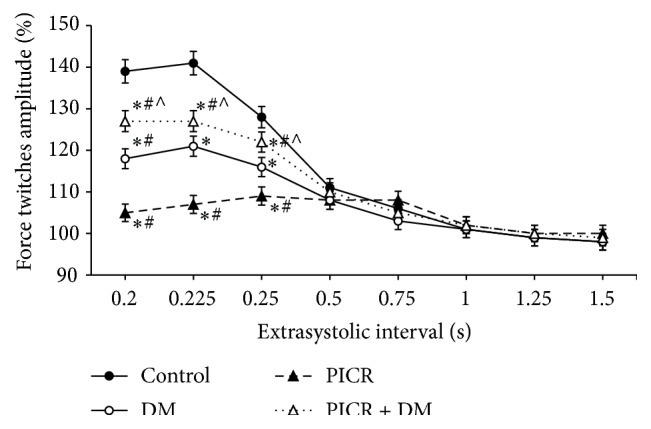
Postextrasystolic contractions of papillary muscles of rats with postinfarction heart failure and diabetes mellitus. Note: ^*∗*^ *p* < 0.001 compared with control, ^#^ *p* < 0.05 compared with DM, and ^∧^ *p* < 0.05 compared with PICR.

It is known that stimulating pulse which falls on the 3rd phase of action potential is not able to induce contractile response. However, it initiates additional income of external calcium ions in the myoplasm. This Са^2+^ is accumulated in SR and takes part in the first PES cycle of contraction-relaxation [22]. For this reason amplitude of PES contraction exceeds amplitude of regular cycle. In our research extraordinary impetus at ES interval of 0.2 s did not cause ES contraction of the myocardium of intact rats (the I group). But we registered 39% increase of PES contraction amplitude compared to the amplitude of regular contraction (Figure 2). With appearance of ES contraction and increase of its amplitude we observed decrease of ES contraction amplitude. For intact animals PES potentiation of contraction was absent on the longest ES intervals (Figure 2).

As we can see from Figure 2 in the II group of rats PES potentiation of contraction of papillary muscles was not observed no matter what the duration of ES interval was. This fact can testify essential decrease of Са^2+^ storing function of SR. Probably, in conditions of postinfarction remodeling of rat myocardium the function of Ca^2+^ transport systems of SR is damaged [11, 28, 29]. While studying the papillary muscles of the rats of the III group, PES potentiation of contraction was essentially lower than in the I group (intact animals) and was 21–16% (Figure 2). In combined postinfarction and diabetic remodeling of myocardium (IV group) on the short ES intervals, the increase in PES contraction was 27–19% (Figure 2). This result testifies maintenance of Са^2+^ storing ability of SR.

It is known that higher activity of peroxidation process is important component of damage of cardiomyocytes due to myocardium infarction [30]. Alteration of the lipid bilayer of membranes with oxygen radicals is considered to be one of the mechanisms of distortion of intracellular Са^2+^ homeostasis and contractile activity of cardiomyocytes. Previously we have shown that higher activity of LPO is also maintained during postinfarction remodeling of heart. Moreover, in simulation of PICR, the dynamics of changes in LPO, the products (TBAAP and DC) in myocardial tissue, and blood serum of rats coincided [31]. On this basis it is possible to define TBAAP and DC concentration in blood serum and to extrapolate it on myocardium.

**Figure 3:**
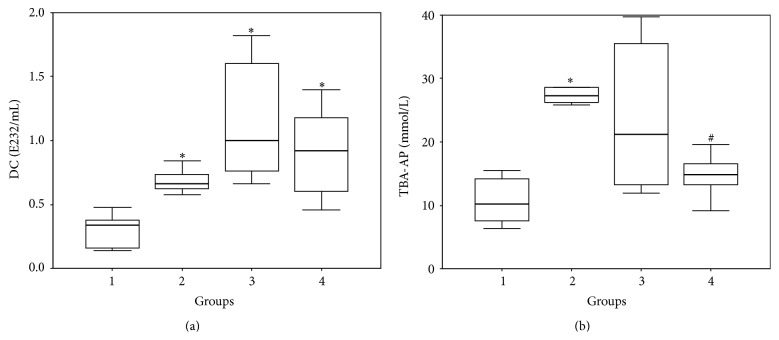
Concentration of DC (a) and TBA-AP (b) in blood plasma of the experimental animals (Me (Q1; Q3)). Note: (1) group: control, (2) group: PICR, (3) group: DM, and (4) groups: PICR + DM. ^*∗*^ *p* < 0.01 compared with group 1 (control) and ^#^ *p* < 0.01 compared with group 2 (PICR).

It is known that activation of free radical oxidation of lipids is also noted at DM [15]. Data obtained at determination of TBAAP and DC concentration in blood serum of animals included in the present research is presented in Figure 3. It can be seen that the PICR animals blood (the II group) contained reliably more LPO products than the intact animal group. Simulation of DM (the III group) also promoted reliable increase of TBAAP and DC concentration. Intensified generation of active oxygen forms and activation of LPO processes at the following pathologies is known fact and is noted in the works of many authors [13–15]. Active oxygen forms in pathologically high concentrations go into reaction and damage both lipids and proteins of cellular membranes and components of blood serum. Literature contains data about decreased activity of proteins and enzymes including Са^2+^-ATPase of cardiomyocytes [13] in pathologies accompanying activation of free radical processes. These results are quite matched with data obtained at estimation of inotropic reaction of papillary muscles of the animals of II and III groups on extrasystolic action. This reaction can be a consequence of decrease in activity of Са^2+^-ATPase and contractile proteins as the result of structural damage caused by active forms of oxygen, violation of lipid bilayer of membrane, and leakage of Са^2+^ from sarcoplasmic reticulum.

Combining development of PICR and DM in theory should cause more manifested LPO activation. However, for animals with combined pathology (the IV group) we obtained paradoxical result. Thus, the value of TBAAP appeared reliably lower than in the II group. Also, the downward trend in DC concentration takes place. Obtained data is well-matched with the results characterizing contractile ability of papillary muscles of the animals of the IV group. Decreased TBAAP concentration testifies the decreased intensity of passing of concluding stages of lipid peroxidation reaction. The fact of negligible decrease in DC testifies that intensity of the first LPO stages remains on sufficiently high level. Metabolites of fat acids forming on these stages can take part in formation of other LPO products [32].

Our data testify that induction of diabetes on the background of postinfarction remodeling paradoxically promotes maintaining functional activity of Са^2+^ transport systems of SR. It may be connected with the fact that glycosylation products increase rigidity of cardiomyocytes membranes on the background of developing hyperglycemia. Enhancement of adaptive reactions at combined development of postinfarction and diabetic damage of myocardium can be connected with peculiarities of intracellular energy metabolism at given pathological states. So, increase of glucose level during the first stages of development of postinfarction cardiosclerosis allows activating glycolysis processes in cardiomyocytes. It is known that positive effect of glucose on the heart functioning in the experimental myocardial ischemia is connected with increase of glycolytic production of ATP [33, 34]. In combination with inhibition of LPO activity, shift of energy metabolism to glycolytic production of ATP can help to obtain higher functional activity of Са^2+^ transport system of SR at combined pathology. Data obtained by us corresponds to the results of other researchers. So, it was shown that ATP which is formed in glycolysis process is the irreplaceable source of energy for Са^2+^ transport system of SR [35]. Increase of ischemic resistivity of myocardium was described for animals with short term of streptozotocin stimulated diabetes in vivo and in vitro [16, 36].

Thus, results of present study showed that in the experimental conditions induction of DM on the stage of formation of postinfarction remodeling increases adaptive ability of myocardium. It is manifested in inhibition of increase in LPO processes activity and maintaining of force-interval reactions of myocardium connected with calcium transport systems of cardiomyocyte SR.

## Figures and Tables

**Table 1:** Body and heart weights of rats after coronary artery occlusion and diabetes induction.

Number	Group	*n*	Body weight, g	Glucose, mol/L	Heart weight/body weight, mg/g	Left ventricle weight/heart weight, mg/mg	Scar area, %
I	Control	12	298 ± 23.7	6 ± 0.37	3.29 ± 0.21	0.645 ± 0.013	—
II	PICR	11	242 ± 11.17^*∗∗*#^	7 ± 0.13^#^	6.27 ± 0.33^*∗∗*#^	0.687 ± 0.016^*∗∗*^	51.3 ± 8.9
III	DM	8	160 ± 14.8^*∗*^	27 ± 2.75^*∗*^	3.77 ± 0.31	0.676 ± 0.014	—
IV	PICR + DM	8	221 ± 4.51^*∗*^	18 ± 1.79^*∗*^	3.37 ± 0.11	0.673 ± 0.019	46.1 ± 2.7

*Note*. PICR: rats with postinfarction cardiac remodeling. ^*∗*^ *p* < 0.01, ^*∗∗*^ *p* < 0.05 compared with control, ^#^ *p* < 0.05 compared with PICR. Scar area was calculated as a percentage from area of free wall of left ventricle.
